# Geospatial Analysis of the Social Determinants of Health of Participants of a Diabetes Management Program to Evaluate Enrollment of Vulnerable Populations

**DOI:** 10.5888/pcd21.240068

**Published:** 2024-08-22

**Authors:** Samantha Kanny, William Cummings, Patricia Carbajales, Janet Evatt, Windsor Westbrook Sherrill

**Affiliations:** 1 Clemson University, Clemson, South Carolina

**Figure:**
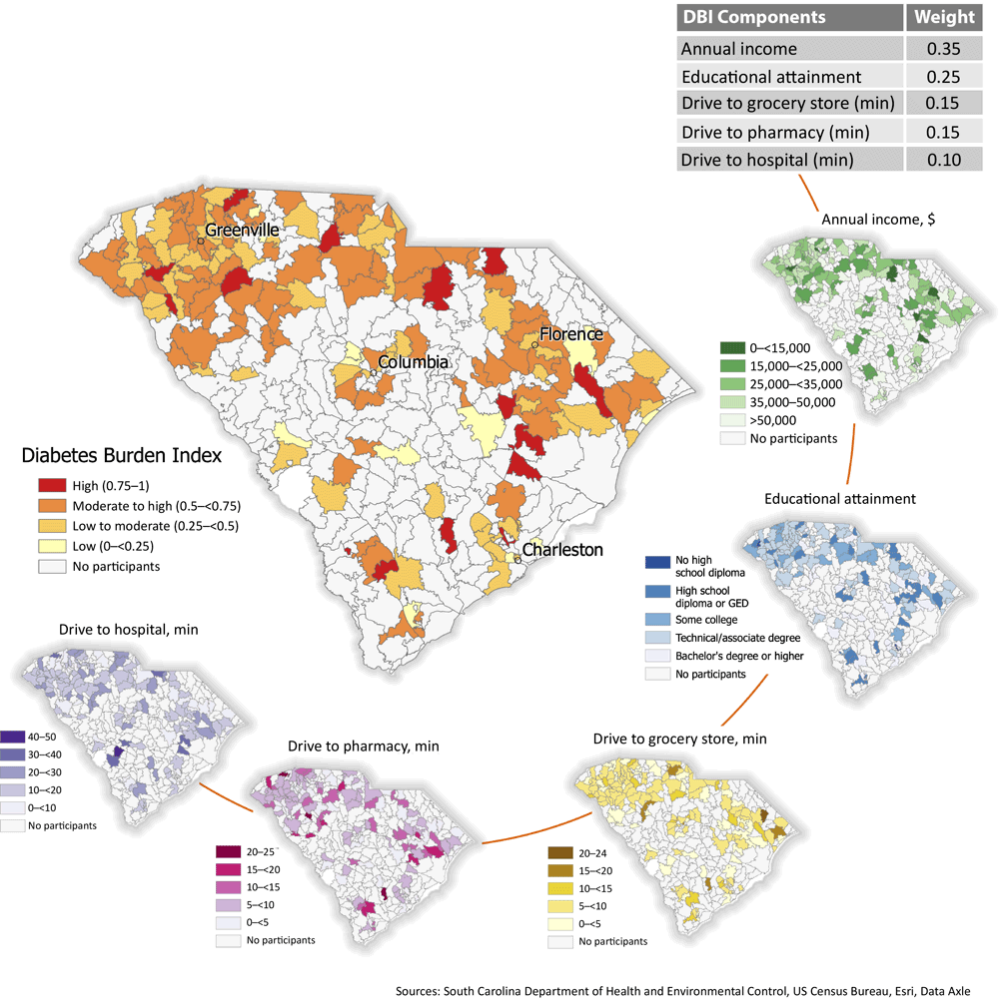
Diabetes Burden Index (DBI), by zip code, South Carolina, October 2023. The DBI evaluates the cumulative burden that HED program participants face in managing their diabetes. Scores range from 0 (lowest burden) to 1 (highest burden). The index considers annual income, educational attainment, and driving time to the closest grocery store, pharmacy, and hospital; components were weighted according to values in the table. Sources: South Carolina Department of Health and Environmental Control, US Census Bureau, Esri, Data Axle. Abbreviations: GED, general educational development; HED, South Carolina Health Extension for Diabetes program.

## Purpose

We used social determinants of health (SDOH) data for people in a diabetes management program to develop a Diabetes Burden Index (DBI) and used geographic information systems technology to show the distribution of the vulnerability of program participants. Literature is lacking that describes the use of mapping to examine SDOH and the burden encountered by people enrolled in chronic condition management programs. Vulnerable populations are at higher risk of developing chronic conditions such as diabetes (1). By understanding the location of at-risk participants, facilitators can increase recruitment of vulnerable individuals. An index such as the DBI can help programs identify populations that are more susceptible to chronic conditions and may also be used to understand participant burden for dealing with chronic conditions, enabling program facilitators to provide extra support to those at risk of attrition.

## Data and Methods

Home addresses and SDOH data, including annual income and educational attainment, were collected from participants in the South Carolina Health Extension for Diabetes (HED) program. For participants who declined to report income, the 2023 per capita annual income for the participant’s census block group was used (2). Hospital locations were provided by the South Carolina Department of Health and Environmental Control and include 108 hospitals licensed as of 2020 (3).

Business locations of grocery stores and pharmacies operating as of 2023 were collected from Esri Business Analyst Business Location Data (4). The closest grocery store, pharmacy, and hospital to each participant was determined by using the facility analysis tool in ArcGIS Pro version 3.1 (Esri). This tool also calculated the optimal driving time between each participant’s address and the nearest facilities.

A weighted composite index, the Diabetes Burden Index (DBI), was developed to evaluate the cumulative diabetes management burden faced by participants. Factors included in the index were based on literature regarding diabetes risk. Socioeconomic status (SES) was selected because the literature supports SES as one of the strongest predictors of diabetes progression and severity, with education and income being the 2 primary socioeconomic indicators (5,6). Drive time to grocery store was chosen as a factor because substantial spatial overlap has been observed between average distance to supermarkets and presence of diabetes; results indicate that the farther an individual lives from a grocery store, the greater the burden of diabetes (7). Drive time to hospital was included in the index because proximity and access to health care facilities, particularly specialty providers with expertise in diabetes, have been observed to affect diabetes management (8,9). Drive time to pharmacy was selected as a factor; the literature indicates that distance from a pharmacy influences the ability to obtain medications, and poor medication adherence results in diabetes complications (10,11). Input on potential weights for each item in the index was solicited from program facilitators, who are most familiar with the challenges faced by participants as well as the influence of associated factors. Analysis of attrition in past cohorts of HED also informed weighting of factors. For each participant, the DBI was calculated as the sum of each weighted component: annual income (weight = 0.35), reported educational attainment (weight = 0.25), and the drive time to the nearest grocery store (weight = 0.15), pharmacy (weight = 0.15), and hospital (weight = 0.10) (Box).

**Box. Weights of Components to Calculate Diabetes Burden Index**

**Table:** 

Component	Weight
Annual income, $	0.35
Educational attainment	0.25
Drive time to nearest grocery store, min	0.15
Drive time to nearest pharmacy, min	0.15
Drive time to nearest hospital, min	0.10

Each participant received a DBI score between 0 and 1 (to be consistent with similar indices), with a score of 0 representing the lowest cumulative burden and a score of 1 representing the highest. DBI scores were classified as low (0 –<0.25), low to moderate (0.25 –<0.5), moderate to high (0.5 –<0.75), and high burden (0.75–1).

For visualization purposes, the DBI was averaged for each zip code serving HED participants. Each of the 5 components of the DBI was also averaged for zip codes so program managers can locate the most vulnerable populations as well as specific variables that influence risk.

## Highlights

A total of 980 participants were included. Most participants enrolled in HED were in the low to moderate DBI range (40% of participants) or the moderate to high DBI range (39% of participants). Additionally, 13% of participants were in the high DBI range. This finding is critical to HED recruitment, as the program aims to serve vulnerable populations.

The diabetes burden in South Carolina is among the highest in the US (12), and the DBI can help identify at-risk populations and highlight locations that are challenging to reach. Although many factors may describe diabetes burden, the 5 included in this index were selected on the basis of input from program partners and problems commonly found in the literature. The DBI is consistent with related literature, which indicates that people with the highest diabetes burden are in areas with the highest diabetes prevalence (13). The DBI is a valuable tool that can accurately identify areas with individuals who face a high burden managing diabetes, inform program recruitment, and direct resources to individuals with the greatest need.

## Action

The findings from this study will be used to determine the primary areas for the Health Extension for Diabetes to increase recruitment of vulnerable populations. This information is key to helping to reduce the diabetes burden in underserved communities in South Carolina. The map can also help to inform HED facilitators’ decision making on future recruitment of vulnerable populations. It will also be used to notify program facilitators about individuals with the highest burden so that appropriate support can be provided. The ability to provide individualized support may help to moderate program attrition.
